# The impact of iron overload and its treatment on quality of life: results from a literature review

**DOI:** 10.1186/1477-7525-4-73

**Published:** 2006-09-28

**Authors:** Linda Abetz, Jean-Francois Baladi, Paula Jones, Diana Rofail

**Affiliations:** 1 Mapi Values Ltd, Adelphi Mill, Grimshaw Lane, Bollington, Cheshire, SK10 5JB, UK; 2 Novartis Pharmaceuticals Corporation, Global Marketing Oncology, 180 Park Avenue, Bldg. 105, Florham Park, NJ 07932-0675, USA

## Abstract

**Background:**

To assess the literature for the impact of iron overload and infusion Iron Chelation Therapy (ICT) on patients' quality of life (QoL), and the availability of QoL instruments for patients undergoing infusion ICT. Also, to obtain patients' experiences of having iron overload and receiving infusion ICT, and experts' clinical opinions about the impact of treatment on patients' lives.

**Methods:**

A search of studies published between 1966 and 2004 was conducted using Medline and the Health Economic Evaluation Database (HEED). Qualitative results from patient and expert interviews were analysed. Hand searching of relevant conference abstracts completed the search.

**Results:**

Few studies measuring the impact of ICT with deferoxamine (DFO) on patients QoL were located (n = 15). QoL domains affected included: depression; fatigue; dyspnoea; physical functioning; psychological distress; decrease in QoL during hospitalization. One theme in all articles was that oral ICT should improve QoL. No iron overload or ICT-specific QoL instruments were located in the articles. Interviews revealed that the impact of ICT on patients with thalassemia, sickle cell disease, and myelodysplastic syndromes is high.

**Conclusion:**

A limited number of studies assessed the impact of ICT or iron overload on QoL. All literature suggested a need for easily administered, efficacious and well tolerated oral iron overload treatments, given the impact of current ICT on adherence. Poor adherence to ICT was documented to negatively impact survival. Further research is warranted to continue the qualitative and quantitative study of QoL using validated instruments in patients receiving ICT to further understanding the issues and improve patients QoL.

## Background

Iron overload is caused by an increased absorption of iron over a long period. Iron overload generally occurs as secondary to conditions that require repeated blood transfusions. Haemoglobinopathies such as, thalassemia and Sickle Cell Disease (SCD), and dysfunctional bone marrow conditions such as Myelodysplastic Syndromes (MDS) are all examples of diseases requiring chronic blood transfusion. If left untreated, iron overload may result in severe morbidity (such as cardiac disease, diabetes, failure of sexual development, osteoporosis, liver damage) and early mortality [1]. However, no immediate iron overload symptoms are present until endocrinopathies and cardiac/organ failure occurs. Given that iron overload occurs gradually, it is likely that the impact on Quality of Life (QoL) will not be noticed until major complications begin to occur in the teens or early twenties for patients with thalassemia and SCD. Patients with MDS face the same problem of gradual development of iron overload with the impact on QoL not noticed until complications begin to occur.

Deferoxamine or desferal (DFO) has until now been considered the treatment choice for patients with chronic iron overload due to blood transfusions. When it is adhered to by patients, it significantly reduces mortality and has an acceptable safety profile. DFO is taken by infusion, often mixed by the patients (or their parents) and takes approximately 5–15 minutes to prepare. The preparation is infused with a thin needle into the arm or abdomen 5 nights per week, for 8 to 12 hours per night [2,3] making it extremely burdensome for the patient. The site of the infusion must be rotated. Pumps are available, with a range in convenience – older pumps tend to be large and noisy, while newer pumps tend to be smaller and quieter. Although DFO is regarded as an effective and well tolerated drug, local injection site reactions that are generally not serious but bothersome to patients include bumps, rashes and bruises, and infections [4,5]. Other side effects experienced by patients on DFO treatment include: neutropenia; haematological toxicity; shortness of breath; headaches; and dizziness [6].

Given the inconvenience and side effects of the treatment, it is likely that iron overload treatment with infusion limits QoL, thereby inhibiting adherence in patients already limited by thalassemia, SCD or MDS [7-10].

Health Related Quality of Life (HRQoL) is a multidimensional concept that represents the patient's overall perception of the impact of illness and its treatment. An HRQoL measure captures at a minimum, physical, psychological (including emotional and cognitive) and social functioning [11]. The measures are seen as methods of capturing patient's opinions and feelings regarding their disease and treatment, their perceived need for healthcare and their preferences for treatment methods and disease outcomes. A recent study in adults with thalassaemia suggested that treatment and cultural differences did not have a major effect on the QoL of patients [12-14]. Pakbaz et al previously suggested that emotional functioning is one of the impaired quality of life domains in patients affected by thalassaemia [15] and a further study showed that thalassaemia patients scored low in their emotional functioning [16].

The objective of this study was to assess the literature for the impact of iron overload and infusion Iron Chelation Therapy (ICT) on patients' QoL and to assess the availability of QoL instruments that have been used with iron overload patients.

## Methods

The methods used to develop hypotheses for the impact of infusion ICT on patients with iron overload is detailed below. It involved a systematic literature review, patient and expert interviews.

### Literature review

We conducted a literature review using electronic databases (Medline and Embase) from 1966 to 2004. The review used a subject and text word search strategy with 'iron chelation', 'thalassemia', 'sickle cell', and 'myelodysplastic syndrome' combined with the terms 'quality of life', 'burden of illness', 'compliance', 'cost', 'cost benefit', 'cost consequence', 'economic evaluation' and 'utility' as the main search terms. In addition, Evidence Based Medicine (EMB) reviews were searched including Cochrane Database of Systematic Reviews (CDSR), American College of Physicians Journal Club (ACP), Database of Abstracts of Reviews of Effects (Dare), and Cochrane Control Trial Register (CCTR).

Further, an additional search was done of the Health Economics Evaluation Database (HEED). For HEED, the phrases 'iron chelation', 'thalassaemia', 'sickle cell', and 'myelodysplastic syndrome' were used.

The review was restricted to English language studies. To satisfy the inclusion criteria, studies had to contain articles that were specific to:

• Iron overload and its treatment in thalassemia, SCD or MDS; or

• Iron overload and QoL research.

Any QoL measures that were found to have been used in the context of patients with iron overload were further researched in the Patient Reported Outcomes Quality of Life International Database (PROQOLID), a database that provides a brief overview of questionnaires used with patients.

### Patient interviews

The interview transcripts of nine patients with iron overload were assessed (4 thalassemia, 1 SCD, and 4 MDS) to determine patients' experiences about the impact of iron overload and its treatment on their daily lives. In addition, four patients (2 thalassemia, 1 SCD, and 1 MDS) participated in a market research study. As part of this study, the patients were asked to provide an overview of the impact of iron overload on their lives. We reviewed the transcripts from these historical interviews in order to gain further insight into how infusion ICT impacts patients' lives.

### Expert interviews

Three iron overload experts representing the UK, US and Italy were interviewed about their observations of the impact of iron overload and its treatment on patients.

## Results

### Literature review

At the onset of this review, 539 abstracts were screened, of which 409 were excluded because of the absence of search terms from either the title or the abstract. In total, 130 articles were reviewed; of which only 15 empirical studies had used validated QoL instruments. Of these, 7 were SCD studies [17-23], 4 were MDS studies [24-27], and 4 were thalassemia studies [2,3,28,29]. All of the evaluated studies focused on the impact of thalassemia, SCD or MDS on patient QoL rather than the impact of ICT on patient QoL. However, mention of the impact on QoL of infusion ICT appears in a number of instances. In one such study, over 50% of patients reported that their activities were often or very often prevented due to DFO treatment and 65% reported dislike of DFO. In addition, 56.2% reported that they would be able to do more things if they did not have to take DFO [3]. In another study, the degree of discomfort associated with DFO treatment was a strong predictor of negative perception of QoL, with the majority on DFO reporting QoL to be fair or poor.

A recurring theme with these patients is the impact of infusion ICT. When these patients were asked what might improve their QoL, the most frequent response concerned the improvement of ICT, particularly the development of an oral drug [28].

Additionally, in another study results indicated that 33% of patients (17 out of 51) with thalassemia or SCD recorded a score of zero in every category of the Sickness Impact Profile (SIP) indicating that some perceived a reduced QoL during DFO therapy [30].

A significant number of anecdotal reports, as well as information derived from clinical experience exist that corroborate our findings regarding the impact of iron overload or ICT on QoL. All the articles reviewed agreed that the infusional (characteristic) of Desferal 5–12 h/d five days/week is a strong impediment of QoL [2,3]. As a corollary, effective oral ICT should improve the QoL of those with iron overload [2,3,17-29]. Further, QoL domains reported as being affected included depression resulting in more hospital visits [20], fatigue, dyspnoea, physical functioning, psychological distress [25], and a general decrease in QoL during hospitalisation [26].

QoL instruments identified from the search included uni-dimensional scales such as the Geriatric Depression Scale (GDS), bi-dimensional scales such as the Hospital Anxiety and Depression Scale (HADS), and multidimensional instruments such as the Medical Outcomes Study Short Form Health Survey 12 items or the 36 items. However, no iron overload-specific QoL instruments were found.

### Results from patient and expert interviews

Since results from the literature review revealed that there were no iron overload specific QoL instruments, hypotheses were generated based on patient and expert interviews in order to develop a disease-specific instrument. Results revealed that the impact of ICT or iron overload on QoL is high but will likely differ by the age of the patient (child, adolescent, young adult, middle aged adult, elderly adult), the length of time on ICT, and by the condition (thalassemia, SCD, MDS).

**Figure 1:**
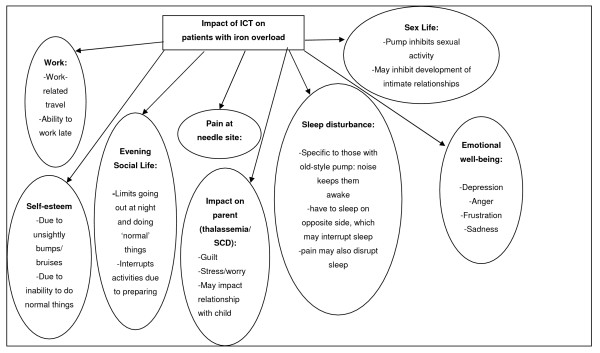
Impact of ICT on patients with IO: Results from patient and clinician interviews.

Figure 1 provides an overview of the hypotheses for the impact of infusion ICT on the QoL of patients with iron overload. In thalassemia, the impact of infusion ICT on QoL is most profound, since patients are required to begin treatment at a very young age (often as young as two or three years old) and continue throughout their life. As a result, the impact on the parent can also be quite high since they have to endure the daily task of inserting a needle into their child and constant battles with their child in order to comply with the treatment regimen, which would then increase parental stress-levels. These battles can carry on from the youngest age through adolescence and therefore may have a negative impact on the parents' relationship with their child and may also cause the child to become over-dependent on their parent. In addition to this, the parent may feel tremendous guilt when they 'give in' to the child's wish not to comply, since they know that the ICT is required to help their child live longer. In addition, as the child reaches adolescence or early adulthood and becomes more in control and responsible for their own treatment, parents may worry if their child is not adequately adhering to the treatment regimen.

The impact of infusion ICT on all patients is high, but it appears that the impact may be greatest for adolescents and young adults (and therefore in thalassemia and SCD), when being able to socialise with peers. How they are perceived by those peers is of paramount importance. The unsightly bumps and bruises caused by infusion ICT appear to most greatly impact adolescents and young adults, with some reporting that they cannot wear certain clothes or are too embarrassed to go out. In young adults and older adults, the impact on work and sex life may also be quite profound. Given that MDS patients tend to be elderly, the most likely impact for active MDS patients would be on evening social life.

When asked about their experiences with infusion ICT, patients stated that the impact of such therapy included pain at the injection site (3/9 [33%]), although one patient reported that the problem was not the pain from the injection site but rather awareness of the pump.

Patients complained of disrupted sleep because of the pump (3/9 [33%]), and that ICT interfered with night-time activities. Further, many patients stated that their sex lives and relationships were affected by their treatment (4/9 [44%]), and some stated that the treatment affected their self-esteem (2/9 [22%]), with some stating that they chose particular clothes to disguise their pumps (2/9 [22%]).

For all patients, satisfaction with DFO was low, primarily owing to local injection-site reactions, inconvenience, and the constraining nature of the therapy. Further, when presented with a hypothetical oral ICT, patients unanimously preferred oral ICT to existing treatment.

## Discussion

Limited empirical studies have assessed ICT's impact on QoL in iron overload patients. However, our results from patient and clinician interviews suggested that the impact of ICT on iron overload patients is profound. Indeed, our research also indicated that QoL impact may inhibit prescription of and adherence to infusion ICT. One study suggested that adherence to ICT is likely to be low given that there is no immediate threat, symptom or impact associated with iron overload (i.e. all effects are long term), a very high impact of infusion ICT on QoL and apparent low satisfaction with infusion ICT [31]. Another study also showed significant iron overload in those who were home transfused indicating poor compliance, though the link with QoL was not established in that study [32].

These findings are significant and provide insight into the impact of iron overload and ICT on patient's daily lives from both the patients' and clinicians' perspectives. The implications of the results suggest that patients are less likely to adhere or continue their treatment regimens as recommended by their doctors. The consequences of non-adherence are significant and may result in severe morbidity (such as cardiac disease, diabetes, failure of sexual development, osteoporosis, liver damage) and early mortality [7].

Further research is warranted to continue the qualitative and quantitative study of QoL using validated instruments in patients with thalassemia, SCD, and MDS receiving ICT, in order to further our understanding of the issues and so improve patients QoL.

It is important to acknowledge that this literature review and its findings are based on published English literature studies which emerged from searching the electronic databases Medline and Embase. Studies were qualitatively discussed, and there was insufficient data to synthesize the evidence. Further research could use statistical methods to explore the net effect of infusion ICT with DFO.

Given that minimal literature was available, we relied on patient and clinician interviews. Some of the patient interviews were historical and the primary purpose of those interviews was not to assess the QoL of the patients, but rather to assess their perceptions of current treatments for iron overload. As a result, in the first instance, we were required to assess the impact on QoL based on the answers to questions that were not specific to QoL. Thus, we may have missed important aspects of life that are impacted by ICT.

In addition, the clinicians interviewed had experience primarily with iron overload in thalassemia and SCD, rather than MDS. As a result, we may have over or under-represented the impact of infusion ICT on MDS patients. We recommend further qualitative and empirical studies to assess the impact of infusion ICT and oral ICT in iron overload patients, using validated QoL instruments to better ascertain the direct impact.

Nevertheless, even with the aforementioned caveats, our results indicated that the impact of infusion ICT on QoL is high. There is a need for easier oral iron chelation therapy that is at least as efficacious and well tolerated in order to improve QoL, increase prescription and adherence rates, and ultimately, reduces morbidity and mortality due to iron overload. Further research should compare DFO with oral chelation therapy according to such endpoints.

## Competing interests

Linda Abetz and Diana Rofail work for Mapi Values, a health outcomes agency. They have worked as advisors for various pharmaceutical companies regarding their clinical trials and patient reported outcomes. Jean-François Baladi and Paula Jones work for Novartis Pharmaceuticals Corporation, USA.

## Authors' contributions

JFB and PJ conceived the study and all authors participated in the design of the study. PJ performed the literature review, and LA, DR and JFB drafted and finalized the manuscript.

## References

[B1] Gabutti V, Borgna-Pignatti C (1994). Clinical manifestations and therapy of transfusional haemosiderosis. Baillieres Clin Haematol.

[B2] Ratip S, Skuse D, Porter J, Wonke B, Yardumian A, Modell B (1995). Psychosocial and clinical burden of thalassaemia intermedia and its implications for prenatal diagnosis. Arch Dis Child.

[B3] Caro JJ, Ward A, Green TC, Huybrechts K, Arana A, Wait S, Eleftheriou A (2002). Impact of thalassemia major on patients and their families. Acta Haematologica.

[B4] Rebulla P (1990). Transfusion reac tions in thalassemia. A survey from the Cooleycare programme. The Cooleycare Cooperative Group. Haematologica.

[B5] Giardina PJ, Grady RW (2001). Chelation therapy in beta-thalassemia: an optimistic update. Seminars in Hematology.

[B6] Alymara V, Bourantas D, Chaidos A, Bouranta P, Gouva M, Vassou A, Tzouvara E, Bourantas KL (2004). Effectiveness and safety of combined iron-chelation therapy with deferoxamine and deferiprone. Hematology Journal 5(6):475-9,.

[B7] Gabutti V, Piga A (1996). Results of long-term iron-chelating therapy. [Review] [109 refs]. Acta Haematologica.

[B8] Porter JB (2001). Deferoxamine pharmacokinetics. Semin Hematol.

[B9] Mourad FH, Hoffbrand AV, Sheikh-Taha M, Koussa S, Khoriaty AI, Taher A (2003). Comparison between desferrioxamine and combined therapy with desferrioxamine and deferiprone in iron overloaded thalassaemia patients. Br J Haematol.

[B10] Piga A (2001). Bone Marrow Transplant.

[B11] Shumaker SA, Berzon RA (1995). The international assessment of HRQL  Theory, translations, measurement and analysis.

[B12] Bowling A, Carr AJ, Higginson IJ and Robinson P (2003). Current state of the art in quality of life measurement. Quality of life.

[B13] Clarke S, Eiser C (2004). The measurement of health-related quality of life (QOL) in pediatric trials: a systemic review..

[B14] Tefler P, Constantinidou G, Andreou P, Christou S, Modell B, Angastiniotis M (2005). Quality of Life in Thalassaemia. Annals of the New York Academy of Science.

[B15] Pakbaz Z, Treadwell M, Yamashita R, Quirolo K, Foote D, Quill L, Singer T, Vichinsky EP (2005). Quality of Life in Patients with Thalassaemia Intermedia Compared to Thalassaemia Major. New York Academy of Sciences.

[B16] Ismail A, Campbell MJ, Ibrahim HS, Jones GL (2006). Health related quality of life in Malaysian children with thalassaemia. Health and Quality of Life Outcomes.

[B17] Anie KA, Steptoe A, Bevan DH (2002). Sickle cell disease: Pain, coping and quality of life in a study of adults in the UK. Br J Health Psychol.

[B18] Cummins O, Anie KA (2003). A comparison of the outcome of cognitive behaviour therapy and hydroxyurea in sickle cell disease. Psychol Health Med.

[B19] Fuggle P, Shand PA, Gill LJ, Davies SC (1996). Pain, quality of life, and coping in sickle cell disease. Arch Dis Child.

[B20] Hasan SP, Hashmi S, Alhassen M, Lawson W, Castro O (2003). Depression in sickle cell disease. Journal of the National Medical Association.

[B21] Palermo TM, Schwartz L, Drotar D, McGowan K (2002). Parental report of health-related quality of life in children with sickle cell disease. J Behav Med.

[B22] Thomas VJ, Hambleton I, Serjeant G (2001). Psychological distress and coping in sickle cell disease: comparison of British and Jamaican attitudes. Ethn Health.

[B23] Thomas VJ, Taylor LM (2002). The psychosocial experience of people with sickle cell disease and its impact on quality of life: Qualitative findings from focus groups. Br J Health Psychol.

[B24] Jansen AJ, Essink-Bot ML, Beckers EA, Hop WC, Schipperus MR, Van Rhenen DJ (2003). Quality of life measurement in patients with transfusion-dependent myelodysplastic syndromes. Br J Haematol.

[B25] Kornblith AB, Herndon JE, Silverman LR, Demakos EP, Odchimar-Reissig R, Holland JF, Powell BL, DeCastro C, Ellerton J, Larson RA, Schiffer CA, Holland JC (2002). Impact of azacytidine on the quality of life of patients with myelodysplastic syndrome treated in a randomized phase III trial: a Cancer and Leukemia Group B study. Journal of Clinical Oncology.

[B26] Sekeres MA, Stone RM, Zahrieh D, Neuberg D, Morrison V, De Angelo DJ, Galinsky I, Lee SJ (2004). Decision-making and quality of life in older adults with acute myeloid leukemia or advanced myelodysplastic syndrome. Leukemia.

[B27] Silverman LR, Demakos EP, Peterson BL, Kornblith AB, Holland JC, Odchimar-Reissig R, Stone RM, Nelson D, Powell BL, DeCastro CM, Ellerton J, Larson RA, Schiffer CA, Holland JF (2002). Randomized controlled trial of azacitidine in patients with the myelodysplastic syndrome: a study of the cancer and leukemia group B. J Clin Oncol.

[B28] Arboretti R, Tognoni G, Alberti D, Thalassarmia ICG (2001). Pharmacosurveillance and quality of care of thalassaemic patients. A large scale epidemiological survey. European Journal of Clinical Pharmacology.

[B29] Smiley M (1986). Beta thalassaemia in Papua New Guinea. Ann Trop Paediatr.

[B30] Basran RK, Fasson FF, Shaw D, Olivieri NF (1994). Assessment of the relative quality of life in patients receiving subcutaneous deferoxamine and the orally active iron chelating agent L1. Blood.

[B31] Cappellini MD, Cohen A, Piga A, Bejaoui M, Perrotta S, Agaoglu L, Aydinok Y, Kattamis A, Kilinc Y, Porter J, Capra M, Galanello R, Fattoum S, Drelichman G, Magnano C, Verissimo M, Athanassiou-Metaxa M, Giardina P, Kourakli-Symeonidis A, Janka-Schaub G, Coates T, Vermylen C, Olivieri N, Thuret I, Opitz H, Ressayre-Djaffer C, Marks P, Alberti D (2005). A Phase III study of deferasirox (ICL670), a once-daily oral iron chelator, in patients with {beta}-thalassemia. Blood.

[B32] Poole JE, Cohn RJ, Roode H, Spector I (1989). Beta-thalassaemia--the Johannesburg experience. S Afr Med J.

